# An overview of the NASA Adaptation and Response in Drylands field experiment scoping study

**DOI:** 10.1017/dry.2025.10006

**Published:** 2026-05-26

**Authors:** Andrew F. Feldman, Sasha C. Reed, Konrad Wessels, Dennis Ojima, David J.P. Moore, William K. Smith, Niall Hanan, Cibele Amaral, Flurin Babst, Joel A. Biederman, Marcy Litvak, Natasha MacBean, Benjamin Poulter, Russell L. Scott, Alicja Babst-Kostecka, Julia K. Green, Raymond F. Kokaly, Ryan Pavlick, Robert Swap, Shawn P. Serbin, Compton J. Tucker, Lixin Wang, Jennifer Watts, Alejandro Flores, James Rattling Leaf, Robert A. Washington-Allen, Karen Prentice, Emily Kachergis, Julian Reyes, Jasmine Ryan, Michael D. SanClements, Henry W. Loescher, Allison K. Leidner, Tyson Swetnam

**Affiliations:** 1 NASA Goddard Space Flight Center, USA; 2 University of Maryland College Park, USA; 3 Southwest Biological Science Center, United States Geological Survey, USA; 4 George Mason University, USA; 5 Colorado State University, USA; 6 The University of Arizona School of Natural Resources and the Environment, USA; 7 New Mexico State University, USA; 8 University of Colorado Boulder, USA; 9 USDA-ARS Southwest Watershed Research Center, USA; 10 The University of New Mexico Department of Biology, USA; 11 Western University, Canada; 12 The University of Arizona Department of Soil Water and Environmental Science, USA; 13 U.S. Geological Survey, USA; 14 NASA, USA; 15 Indiana University Indianapolis, USA; 16 Woodwell Climate Research Center, USA; 17 Boise State University, USA; 18 University of Nevada Reno, USA; 19 Bureau of Land Management, USA; 20 Battelle, USA; 21 US Department of the Interior, USA

**Keywords:** drylands, ecology, ecosystems, ecosystem services, dryland ecosystems, remote sensing, field campaign

## Abstract

Drylands cover 41% of Earth’s land surface, support 36% of the global population and contribute 60% of global food production. Despite these ecosystems’ importance and high vulnerability to droughts and heatwaves, drylands remain some of the most understudied systems on Earth. Monitoring drylands is challenging due to their complex ecosystem structure of visible soil mixed with diverse plant species that respond rapidly to weather and climate. In 2023 and 2024, a NASA scoping study was conducted for a proposed dryland terrestrial ecology field campaign called Adaptation and Response in Drylands (ARID). Thereafter, the NASA ARID scoping team submitted their campaign proposal to NASA Headquarters, providing a study design for how field, aircraft and satellite measurements, as well as modeling, could address the most critical fundamental and applied science questions in drylands. The extensive strategic vision was created by and for the drylands research community, including remote sensors, modelers, experimentalists and ecologists from across the world, and the overall approach can be further utilized and altered for different uses and data information needs. Here, we summarize the final ARID research agenda, including its main objectives, field campaign strategy, data end-user support strategy, and U.S. and global community engagement.

## Impact statement

Drylands are water-limited ecosystems, which cover 41% of Earth’s land surface, while contributing a substantial amount of our food supply. They are also some of the most vulnerable to weather extremes and change. Despite their importance, their soil and plant behavior across a range of conditions is not well understood, making it challenging to predict a range of human-relevant processes like crop and rangeland production and wildfire and flood risk. The NASA Terrestrial Ecology program funded a 1.5-year planning effort for a potential field campaign focused on drylands called Adaptation and Response in Drylands (ARID). The ARID team was tasked with identifying research and resource management needs as well as a preliminarily plan for the campaign. The ARID scoping study was comprehensive in including input from an array of interested parties, including research scientists, land managers and decision makers, and Tribal Nations within the United States (U.S.) and globally. Consequently, the proposed campaign focuses on advancing science goals related to water, carbon and ecosystem dynamics while also addressing land management goals, such as those of ranchers and farmers. The ARID campaign plan, summarized here, also lays the foundation for future campaigns to advance understanding and management of Earth’s resources and ecosystems.

## Introduction

Drylands play vital roles in the Earth System and human prosperity. These water-limited ecosystems represent the planet’s largest terrestrial biome, covering 41% of the Earth’s land surface (and 45% of the United States) and supporting the livelihoods of roughly 2.7 billion people (or 36% of the global population) (Safriel et al., 2005; Reynolds et al., 2007; Wang et al., 2022). Drylands contain 25% of cultivated lands and 65% of range or grazing land (Safriel et al., 2005), consequently sustaining 60% of global food production (Wang et al., 2022). They are also home to 15 of Earth’s 36 biodiversity hotspots, contain strategic mineral and energy resources, and dominate the trend and interannual variability of the global terrestrial carbon sink (Poulter et al., 2014; Cartereau et al., 2023; Gross et al., 2024; Sitch et al., 2024). Overall, drylands are estimated to provide about $18.4 trillion/year in ecosystem goods and services (Costanza et al., 2014).

Many drylands show dramatic sensitivity to weather and extreme events, such as El Niño Southern Oscillation (ENSO), droughts, heatwaves, floods and fire (Holmgren et al., 2006; Biederman et al., 2016; Huang et al., 2016; Maestre et al., 2016; Berdugo et al., 2020). For example, in the western U.S., hot and dry conditions since the early 2000s and recent heatwave events have caused extensive plant mortality, with significant impacts on dryland ecosystem structure and function (Allen et al., 2010; Moore et al., 2016; Dannenberg et al., 2021). This has negative implications for societal well-being, threatening farming and ranching livelihoods, and leading to crises related to poverty, conflict and human migration (Abel et al., 2021). Evidence is mounting that dryland plant communities around the world are already changing rapidly, with woody encroachment into grasslands and savannas, exotic grass invasions, intensified fire regimes and large-scale ecosystem mortality events (Allen et al., 2010; Jones et al., 2020). These changes, in turn, create challenges, especially for rangelands, which are predominantly located in drylands and which constitute the world’s largest land use. Specifically, rangelands are experiencing significant changes to vegetation community composition (e.g., from shrub encroachment and exotic grass invasion), with a loss of biodiversity and forage productivity with critical implications for livestock production, food security and the support of human livelihoods (Godde et al., 2021; Maestre et al., 2022; Cartereau et al., 2023).

Drylands are one of the least understood terrestrial biomes despite their large human population, contributions to agricultural production and land coverage (Schimel, 2010; Smith et al., 2019). In part, this poor understanding stems from the fact that these ecosystems are challenging to evaluate because they are highly heterogeneous in space and time. Spatially, there is high plant diversity interspersed amongst patches of bare soil, bedrock and biological soil crusts (a photosynthetic soil community made up of mosses, lichens and/or cyanobacteria that represent a common dryland cover type) (Maestre et al., 2016; Tucker et al., 2023). In addition to having high spatial heterogeneity, considering temporal dynamics, drylands respond rapidly to stochastic rainfall events and extremes like droughts, floods and heatwaves (Noy-Meir, 1973). Hence, satellite remote sensing systems and core algorithms have not always been designed for the high spatial and temporal heterogeneity characteristic of dryland environments, and in many cases are thus unable to represent and monitor spatial or temporal dynamics with sufficient detail (Li et al., 2008; Smith et al., 2019). This information gap can greatly reduce the utility of these data for a wide range of U.S. and global decision-makers (Noy-Meir, 1973; Allred et al., 2022). Similarly, current ground observation networks that monitor vegetation and soil dynamics, water cycling and biogeochemistry remain sparse and inconsistent in drylands compared to more mesic systems. Consequently, our ability to parameterize predictive models is limited by an inadequate representation of dryland above- and belowground carbon, water and energy dynamics with existing information (Schimel, 2010; MacBean et al., 2021). Moreover, our relatively low capacity to model drylands complicates our ability to manage their resources, predict their recovery from wildfire and other disturbances, reduce the extent and impact of invasive species and support associated agricultural and livestock economies.

Importantly, new remote sensing, modeling and ground-based techniques offer an opportunity to address these challenges, providing an unprecedented opportunity to better assess, scale, forecast and make decisions for the dryland biome. Now is an ideal time for an improved predictive understanding of how drylands function, their potential feedbacks to a changing environment, the myriad societal benefits they provide and the decisions that can sustain these vital ecosystems into the future. We aim to facilitate the comprehensive exploration of these dryland research challenges and opportunities as part of a proposed dryland terrestrial ecology field campaign called Adaptation and Response in Drylands (ARID) (Feldman et al., 2024; Reed et al., 2025). Over a multi-year to decade period, the goal of the ARID campaign is to substantially advance our understanding, monitoring and modeling of drylands by jointly using in-situ, airborne and satellite observations. Transformative research and collaboration can be used to inform action that helps reduce human and ecosystem vulnerability. Therefore, ARID aims to make actionable information available for dryland residents, land managers and policy-makers. Accordingly, ARID was developed with the engagement and input of dryland scientists and resource managers from the U.S. and globally, thus co-producing the research plan to ensure that the science is useful for decisions and management actions across scales.

This article serves to briefly describe the main components of the final proposed ARID field campaign plan and agenda completed in December 2024 based on a scoping process that took place between 2023 and 2024. Here, we seek to facilitate the use and accessibility of the co-developed research road map by describing ARID’s core goals, field campaign strategy, practitioner support strategy and community engagement. This article complements Feldman et al. (2024), which introduced ARID and described the community engagement strategy. The final ARID scoping report is available online via a data repository (Reed et al., 2025).

### ARID campaign core goals

Over the past four decades, NASA’s Terrestrial Ecology Program field campaigns have focused on temperate ecosystems, tropical forests and most recently arctic and boreal systems (Arctic Boreal Vulnerability Experiment; ABoVE) (Miller et al., 2019). These efforts have demonstrated the power of coordinated field campaigns to vastly advance our understanding of complex systems, to build long-lasting collaborations and to train the next generation of Earth scientists. Although global authorities recognize the crisis that changes in global drylands signify for social-ecological systems, there has not yet been a large-scale, sustained research campaign targeting dryland ecosystems. Such a campaign would robustly fill this critical knowledge gap.

Sustained, multi-year research in heterogeneous dryland ecosystems requires an understanding contextualized within larger planetary-scale dynamics. Short-term or single-site studies can miss broader patterns and thereby miss a key opportunity to inform sustainable policies and management decisions. In addition, a coordinated campaign focused on drylands would enable, facilitate and foster new technological developments that are needed to improve management and monitoring of at-risk natural resources in dryland environments.

ARID is a proposed NASA Terrestrial Ecology field campaign that is poised to transform our ability to assess, predict and inform effective decision-making for dryland ecosystems (https://aridscoping.arizona.edu). Throughout 2023 and 2024, the ARID team conducted a scoping study and wrote a final report that provided a research road map and field implementation plan for consideration by NASA’s Terrestrial Ecology Program (Feldman et al., 2024; Reed et al., 2025). It assembled a prioritized research agenda and an initial implementation plan consistent with the mandate of NASA Earth Science Division’s Integrated Earth System Observatory (iESO) to “*create a 3D, holistic view of Earth, from bedrock to atmosphere.*” (Greene and Fox, 2021). Across the western U.S. and several international locations, ARID plans to coordinate the integration of data from satellites, aircraft, ground-based observations and advanced modeling. In doing so, ARID aims to enable the research and land management communities to significantly advance knowledge, model representation and management options for these vast and vulnerable landscapes. From its inception, ARID’s research agenda has been based on extensive engagement with the scientific community and directly informed by a wide range of end-users.

ARID’s core objective is to help reduce human and ecosystem vulnerability to environmental extremes and change in drylands through transformative research and innovative collaborations. Recently deployed satellite instruments now make measurements at or close to the spatial and temporal resolutions needed to assess and scale dryland structure and function across the globe. ARID seeks to support the research community in the use of cross-scale, multi-disciplinary approaches to address four science themes aligned with key knowledge gaps, with associated research questions:

1. *Climate Variability and Drought: How are climate extremes like droughts, heatwaves and large rain pulses impacting dryland systems, and how do they interact with changing fire regimes, land cover change and land-atmosphere interactions?*
2. *Ecosystem Structure, Function and Biodiversity: What are the main mechanisms driving the spatiotemporal distributions of dryland structure, function and biodiversity, and what is their vulnerability to change?*
3. *Carbon Cycle Interannual Variability and Long-Term Trends: What is the contribution of drylands to the mean, trend and interannual variability of global terrestrial carbon uptake, what drives these patterns and what can we expect from them in the future?*
4. *Social-Ecological Systems: What are the consequences of changes in drylands for social-ecological systems, and what management (e.g., mitigation and adaptation) solutions can maintain the critical services provided by drylands even in the face of change?*

The first three science themes focus on the fundamental interactions between drivers of change, ecosystem responses and feedback mechanisms. The fourth theme focuses on human systems and land management. Taken together, ARID embraces drylands as multi-faceted, complex systems, meriting broad interdisciplinary approaches, including terrestrial ecology, hydrology, biogeochemistry, remote sensing and modeling.

### ARID field campaign strategy

**Figure 1.**
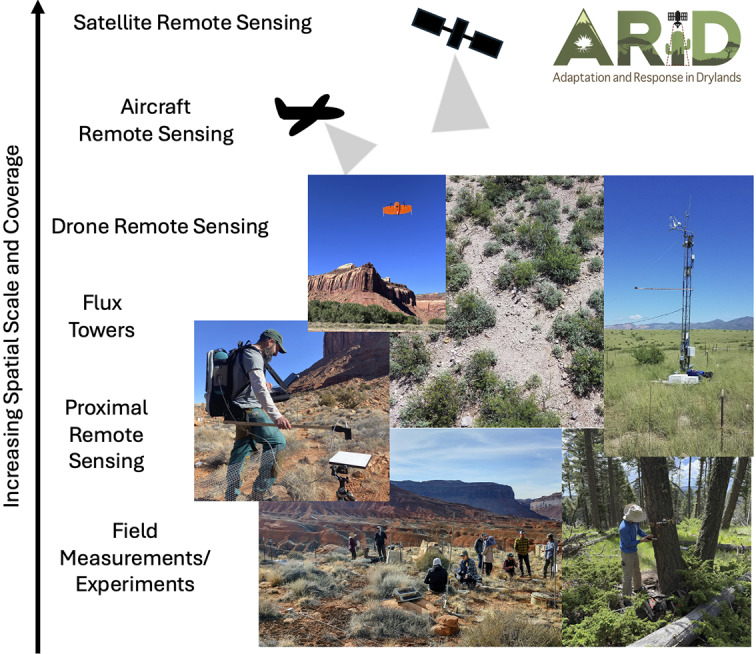
Adaptation and Response in Drylands (ARID) field implementation strategy for observing drylands’ spatiotemporally heterogeneous landscapes through multi-scale field, aircraft and satellite measurements and coupling to models. ARID will blend cross-scale spatial and temporal measurements to advance our understanding, predictive capacity and management options for drylands, as well as improve the tools available to assess these dynamic ecosystems. The three left-most photographs were taken at a high desert experimental facility near Moab, Utah, USA, where drone, airplane, satellite and ground-based data were collected simultaneously. The top-left photograph shows a fixed-wing drone, the middle-left photograph shows ground-based proximal remote sensing and the lower-left photograph shows a team of dryland scientists collecting data and samples on the ground. The upper-middle photograph was taken by drone at Walnut Gulch Experimental Watershed in Lucky Hills, Arizona, USA. The upper-right flux tower photograph was taken at the Kendall Grasslands AmeriFlux site near Tucson, Arizona, USA. The bottom-right photograph shows a dryland forest in the Valles Caldera near Sante Fe, New Mexico, USA. Photographs are owned and provided by the authors. Figure 1. long description.

The ARID campaign aims to collect data across scales, from space, the air and on the land, using a suite of available observational approaches, including field- and tower-based, drone-based, airborne and satellite remote sensing observations (Figure 1). The goal is to integrate these observations and advance multi-scale modeling. As examples of observational assets from NASA, ARID will leverage a suite of current spaceborne sensors, such as ECOSTRESS, EMIT, SMAP and Landsat, and recently launched and future ones, including NISAR. Use of other spaceborne assets from the program of record, including commercial sources and those from non-U.S. agencies, could also inform observations and modeling. Data from spaceborne sensors are planned to be combined with those from NASA airborne assets (e.g., AVIRIS, G-LiHT, SLAP, UAVSAR, MASTER, etc.), as well as drone and ground-based measurements (Figure 1). Ground and aircraft measurements will also be used to improve our quantitative and predictive understanding of drylands via calibrating and validating satellite measurements, as well as via developing relationships (through next-generation machine learning and other statistical techniques) between surface properties and remote sensing measurements to scale up and forecast these properties.

A guiding principle of the field campaign is to leverage and complement existing in-situ research and monitoring investments, providing an opportunity to address research needs with benefits for science, societies and economic vitality in drylands. Successful coordination of these efforts will fill in the gaps between what satellites observe and what is happening on the ground, leading to more accurate observations, scaling approaches, understanding and models. More broadly, ARID’s scoping study and future implementation will provide a blueprint for future satellite missions and field campaigns to improve our monitoring and understanding of drylands.

The core activities during ARID are proposed for the western U.S., taking advantage of natural gradients of aridity, elevation and vegetation, as well as a wealth of regional practitioner knowledge and data end-user interest. The U.S. domestic work is organized into geographic focal areas, including the Southwest U.S., the Western Great Plains, the Mountain West and the Great Basin. Within this domestic focus, we aim to leverage and complement existing NASA data, partner airborne campaigns and existing ground datasets and observation networks, including other federal investments (e.g., AmeriFlux, NEON, LTER, BLM Assessment, Inventory and Monitoring, National Park Service Inventory and Monitoring, USDA Forest Inventory and Analysis, USGS GEMx). This approach provides an opportunity for ground-truthing and mechanistically linking ground-based observations with remotely sensed data to generate new understandings of dryland systems. In this way, ARID would provide a nexus of collaboration among existing institutions, universities, agencies, researchers and end-users who require the complex cross-scale data and observations that a coordinated leaf-to-orbit dryland field campaign would provide.

While these U.S. focal areas collectively represent a large portion of the aridity range found across global drylands, international sites are necessary to address scientific questions at a global scale and to represent the wide array of dryland climates, biota and land-use types. The global expertise on drylands and the research infrastructure and data needs also provide powerful opportunities to further understand and make decisions for these widely distributed systems. Candidate international sites were selected based on community input, assets of local partners (like long-term field data), gradients of key variables and ecosystem types needed to test core hypotheses, logistical feasibility, dependability of plans and safety and security. Candidate sites are in southern Africa, northern Mexico, Australia, and the Cerrado and Caatinga regions of South America. Many of ARID’s leading international partner institutions (such as TERN in Australia and SAEON in South Africa) are members of Global Ecosystem Research Infrastructure (GERI), together with the U.S.’s NEON, where they coordinate research and international network building on global issues such as ecological drought (Loescher et al., 2022).

Guiding principles to implement the ARID campaign operationally are to utilize ground, aircraft and spaceborne instruments (primarily from NASA, but the sources of spaceborne data can be expanded) as well as the data they produce to: (i) ensure observations and modeling are at scales appropriate to capture the spatio-temporal variability and cross-scale feedbacks of drylands, (ii) leverage existing assets (data and infrastructure) and investments by partners, (iii) capitalize on existing national and international networks, including the use of “super sites” for coordinated, synergistic field data collection, (iv) use multi-temporal airborne and unmanned aircraft system (UAS) acquisitions, (v) support ongoing and upcoming NASA missions and (vi) balance operational risk with scientific reward. We aim to apply ARID’s multidisciplinary approach at locations that are logistically feasible and safe for researchers, balancing a focus on U.S. sites while also addressing international priorities. ARID’s concept of operations is modular and can be phased; the logistically straightforward U.S. study regions can be implemented first, while more complex international campaigns can be carefully planned and phased in later. By leveraging new and upcoming remote sensing tools, existing research networks and strong research-management partnerships, ARID can build a critically needed actionable understanding of drylands.

### Data end-user and practitioner support

ARID seeks to support data end-user and practitioner goals in a manner fully aligned with NASA’s Earth Science to Action (ES2A) strategy, which has a goal of innovating and collaborating across research and end-user sectors (St. Germain et al., 2024). The ARID field campaign proposes supporting algorithm development and uncertainty estimation of fundamental remote sensing products that feed directly into existing workflows of agencies, and potentially into processes of private land and water managers. The ARID field campaign is the embodiment of ES2A as it both directly addresses science questions and informs applications and adaptation strategies for federal agencies, Tribal authorities and land managers more broadly. The NASA ARID campaign can thus deliver actionable information and insights to many U.S. land managers and a suite of national and international end-users. In collaboration with NASA data infrastructure capabilities, ARID intends to use cyberinfrastructure to create a unified virtual hub for access to ARID products, including open data, software and publications. The proposed cyberinfrastructure plan would enable data processing and analytics, promoting collaborative team science and use by the broader community.

Upholding these principles, ARID focuses substantially on the U.S. due to the strong national need for actionable dryland science. Understanding U.S. drylands is of particular importance in order to inform national resource management and security, as drylands cover ~83% of the western U.S. Currently, drylands are under prolonged water stress from the ongoing North American megadrought (Williams et al., 2022). These climate extremes can affect activities at the U.S. Department of Defense, U.S. Bureau of Land Management (BLM), U.S. Department of Agriculture (USDA) Forest Service, National Park Service, Fish and Wildlife Service, Bureau of Reclamation and Tribal Nations, where together they manage >2 million km^2^ of drylands for multisectoral land use (rangeland, energy, mining, cultivation, forestry, conservation). Most of this land experiences ongoing changes in vegetation and productivity, shrub encroachment and invasive grasses, loss of grazing resources and changes in fire regimes, placing it under further risk of large-scale instability and shift in ecosystem services (Godde et al., 2021). The BLM alone manages almost 10% of U.S. land area, with a dominant proportion in drylands. Additionally, land management and conservation efforts are also incentivized on private lands, for example, through the USDA Natural Resource Conservation Service (Watts et al., 2025). Thus, an improved understanding of drylands could have a massive management impact across a range of agencies, institutions and entities. Moreover, alternative energy siting and nature-based climate solutions (NbCS) programs frequently target drylands for development, restoration and/or afforestation to enhance carbon sequestration and the human environment. However, there is a lack of understanding of the critical tradeoffs for biodiversity, water resources, surface energy balance and social-ecological feedbacks associated with these solutions in drylands. The tools and scalable information to evaluate these actions and their consequences have historically been limited. The ARID field campaign intends to directly address this critical knowledge gap.

### Research team implementation

ARID is designed to be conducted through a range of funding models. Based on the examples of former NASA Terrestrial Ecology field campaigns, ARID could be funded through efforts within research teams and directed by NASA Headquarters, with the majority of the funding being made available to the larger research community. Research teams of varying sizes could propose research to NASA through competitive solicitations and potentially other agencies to address ARID’s defined goals. For cases of applied land management research, these teams would include end users to accomplish specific societal goals. Aircraft measurements could be directly funded by NASA to provide critical data for the research teams. A potential limitation with this funding model is the lack of interconnectedness among project teams, and thus advances would be made on a range of dryland questions, but with potentially limited interconnection of findings. To help mitigate this issue, ARID’s strategic plan includes directed modeling efforts with continuous funding throughout the campaign to collate efforts and better connect research efforts. Other funding models will be explored as ARID is implemented.

### Community engagement

**Figure 2.**
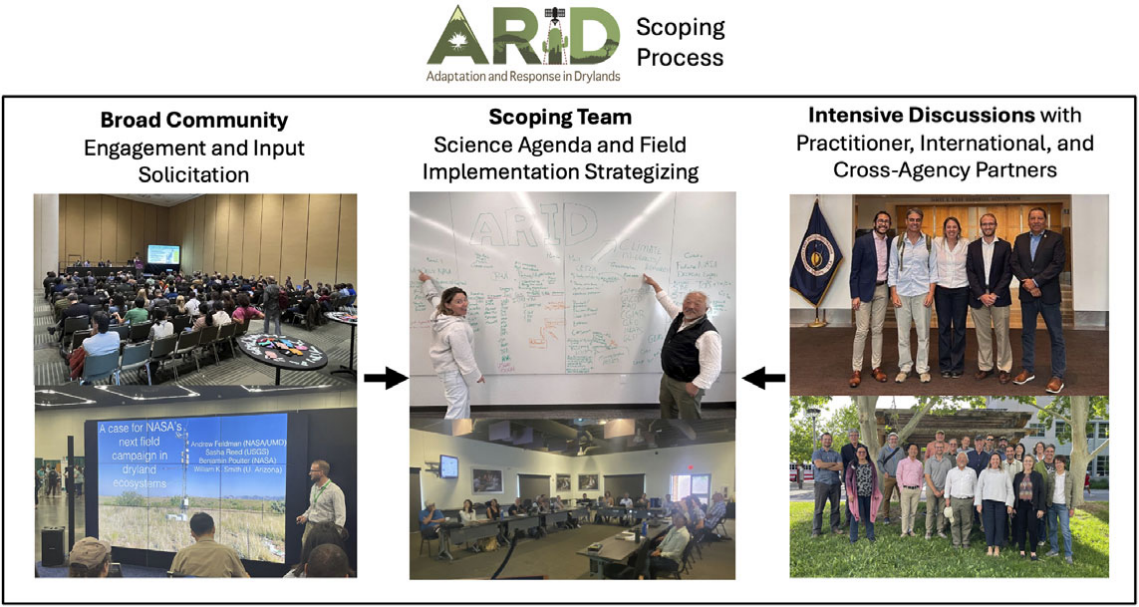
The scoping process included both broader sessions soliciting community input and intensive discussions with cross-agency, practitioner and international partners. Both forms of discussion drove the ARID scoping team’s synthesis of a dryland science agenda and field implementation strategy. Photographs are from the American Geophysical Union and ESA annual meetings (left), ARID strategy and scoping workshops in Albuquerque, NM, USA and Tucson, AZ, USA (center) and intensive discussions in Washington, DC, USA and Albuquerque, NM, USA (right). Photographs are owned by the authors. Figure 2. long description.

ARID’s planning was built upon the co-development and knowledge-sharing frameworks from over 160 community engagement events (Feldman et al., 2024). This included broad feedback from conference town halls and webinars as well as intensive discussions through workshops, technical working group meetings, roundtables, in-person visits and Tribal meetings (Figure 2). For example, the ARID scoping team held workshops in Arizona (October 2023), New Mexico (May 2024) and Washington D.C. (July 2024) that brought together researchers, partner organizations, end-users, Tribal Nations and federal agencies (Feldman et al., 2024). The ARID scoping team and working groups used this input to develop and refine the ARID research agenda and campaign strategy (Figure 2). We highlight three key communities engaged during ARID’s co-development process, as they were particularly important in shaping ARID’s vision. First, the ARID team worked directly with land managers and decision makers responsible for the stewardship of domestic natural resources. Their expertise and clear vision of data needs were pivotal for informing a research road map that will provide information that is useful, usable and used. Second, ARID’s leadership by and engagement with Tribal groups established an ongoing means to co-develop data to meet their community needs and share knowledge. Their expertise, ideas and opportunities for impactful research were critical in shaping ARID’s science, training and community-building strategies. Finally, ARID included international science partners who have committed formal support to the ARID project. The inclusion of international sites enables addressing research questions that require understanding the range of conditions experienced across global drylands. Through ARID’s co-design process, we captured a range of community input and strategically secured multi-sectoral partnerships essential for developing an ambitious and relevant dryland research agenda. This approach to join a range of expertise not only boosts the chances of scientific advancement in drylands but also builds the next cohort of scientists and decision-makers prepared to address current and future issues in drylands.

## Conclusions and expected outcomes

If conducted over a decadal timespan with extensive investment by the research community, ARID will provide important opportunities to:

- *Advance* our foundational understanding of how drylands function and contribute to the U.S. and the Earth System
- *Transform* our capacity to monitor and predict food production, vegetation structure and composition, water availability and fires with new monitoring techniques and capabilities, which directly informs decision-support for decision-makers
- *Revolutionize* multi-scale, multi-sensor remote sensing of dryland patterns and processes, enhancing our capacity to use current and future NASA satellite observations in drylands
- L*everage* and complement existing investments and research in drylands with the goal of improving the assessment and management options for dryland natural resources
- *Train* the next generation of Earth scientists through multidisciplinary education, training and network building
- *Develop* novel open data products and tools to inform science-based decision-making toward sustaining dryland ecosystems for future generations
- *Build* a collaborative international community focused on interdisciplinary dryland science for the sustained study of dryland ecosystems

The ARID mission arrives at a critical juncture as climate impacts intensify and the need for sustainable management of dryland regions becomes more imperative than ever. While fine-scale spatial variability and rapid temporal dynamics of drylands have significantly challenged prior efforts for a quantitative, scalable understanding of Earth’s drylands, ARID’s tiered observation plan (the integrated combination of using multi-sensor airborne, uncrewed aircraft systems and in-situ data to challenge theory and models; Figure 1) can create new knowledge, fill critical gaps and improve our remote sensing and modeling capabilities. Moreover, our scoping study efforts brought together strong research and educational communities and partnerships that serve as a *de facto* ARID network eager to tackle the complex problems facing drylands and co-create the actionable understanding to help manage these valuable U.S. and global resources for the future. Overall, we argue that a dryland field campaign effort like ARID is a high-priority, low risk and high-return proposition.

Ultimately, ARID serves to substantially build on the range of dryland research being conducted globally while extending beyond former campaigns that were more limited in scope. ARID also endeavors to empower future dryland research that will build upon these research goals, further contributing to ARID’s main research questions and intended outcomes. Finally, the ARID campaign and scoping plan lay the foundation for future campaigns to advance understanding and management of Earth’s resources and ecosystems.

## Data Availability

No data were used in the generation of this manuscript.

## Figure 1. Long description

The schematic is organized vertically with a left-aligned arrow labeled Increasing Spatial Scale and Coverage. From bottom to top, the tiers are Field Measurements/Experiments, Proximal Remote Sensing, Flux Towers, Drone Remote Sensing, Aircraft Remote Sensing, and Satellite Remote Sensing. Each tier has a corresponding photo or icon to its right. At the bottom, Field Measurements/Experiments shows a group of scientists collecting data in a dryland landscape. Above, Proximal Remote Sensing displays a researcher using a backpack-mounted sensor and a ground-based instrument. The Flux Towers tier shows a tall instrumented tower in grassland. Drone Remote Sensing is represented by a fixed-wing drone in flight above red rock terrain. Aircraft Remote Sensing is depicted by a black airplane icon with a downward-facing sensor cone. Satellite Remote Sensing is shown with a satellite icon and sensor cone. The ARID logo is in the upper right. The photos span sites in Utah, Arizona, and New Mexico, illustrating the integration of multi-scale measurements for dryland research.

Navigate back to Figure 1..

## Figure 2. Long description

From left to right, the first panel is labeled Broad Community Engagement and Input Solicitation and contains two photos: the top shows a large audience seated in rows facing a presentation screen, while the bottom shows a presenter standing before a projected slide titled ‘A case for NASA’s next field campaign in dryland ecosystems’ with a list of names and affiliations. The center panel, labeled Scoping Team Science Agenda and Field Implementation Strategizing, contains two photos: the top shows two people standing at a whiteboard covered in handwritten notes and diagrams, and the bottom shows a group of people seated at tables in a meeting room with a screen at the front. The right panel, labeled Intensive Discussions with Practitioner, International, and Cross-Agency Partners, contains two photos: the top shows a group of seven people standing indoors in front of a flag, and the bottom shows a larger group of people standing outdoors on grass with trees and buildings in the background. Black arrows between the panels indicate the process flow from left to center to right.

Navigate back to Figure 2..

## References

[r1] Abel C, Horion S, Tagesson T, Keersmaecker WD, Seddon AWR, Abdi AM and Fensholt R (2021) The human–environment nexus and vegetation–rainfall sensitivity in tropical drylands. Nature Sustainability 4(1), 25–32. 10.1038/s41893-020-00597-z

[r2] Allen CD, Macalady AK, Chenchouni H, Bachelet D, McDowell N, Vennetier M, Kitzberger T, Rigling A, Breshears DD, Hogg EH, Gonzalez P, Fensham R, Zhang Z, Castro J, Demidova N, Lim JH, Allard G, Running SW, Semerci A and Cobb N (2010) A global overview of drought and heat-induced tree mortality reveals emerging climate change risks for forests. Forest Ecology and Management 259(4), 660–684. 10.1016/j.foreco.2009.09.001

[r3] Allred BW, Creutzburg MK, Carlson JC, Cole CJ, Dovichin CM, Duniway MC, Jones MO, Maestas JD, Naugle DE, Nauman TW, Okin GS, Reeves MC, Rigge M, Savage SL, Twidwell D, Uden DR and Zhou B (2022) Guiding principles for using satellite-derived maps in rangeland management. Rangelands 44(1), 78–86. 10.1016/j.rala.2021.09.004

[r4] Berdugo M, Delgado-Baquerizo M, Soliveres S, Hernández-Clemente R, Zhao Y, Gaitán JJ, Gross N, Saiz H, Maire V, Lehman A, Rillig MC, Solé RV and Maestre FT (2020) Global ecosystem thresholds driven by aridity. Science 367(6479), 787–790. 10.1126/science.aay5958 32054762

[r5] Biederman JA, Scott RL, Goulden ML, Vargas R, Litvak ME, Kolb TE, Yepez EA, Oechel WC, Blanken PD, Bell TW, Garatuza-Payan J, Maurer GE, Dore S and Burns SP (2016) Terrestrial carbon balance in a drier world: The effects of water availability in southwestern North America. Global Change Biology 22(5), 1867–1879. 10.1111/gcb.13222 26780862

[r6] Cartereau M, Leriche A, Médail F and Baumel A (2023) Tree biodiversity of warm drylands is likely to decline in a drier world. Global Change Biology 29(13), 3707–3722. 10.1111/gcb.16722 37060269

[r7] Costanza R, de Groot R, Sutton P, van der Ploeg S, Anderson SJ, Kubiszewski I, Farber S and Turner RK (2014) Changes in the global value of ecosystem services. Global Environmental Change, Elsevier Ltd 26(1), 152–158. 10.1016/j.gloenvcha.2014.04.002

[r8] Dannenberg MP, Smith WK, Zhang Y, Song C, Huntzinger DN and Moore DJP (2021) Large-scale reductions in terrestrial carbon uptake following central pacific El Niño. Geophysical Research Letters, 48(7), 1–11. 10.1029/2020GL092367

[r9] Feldman AF, Reed S, Amaral C, Babst-kostecka A, Babst F, Biederman JA, Devine CJ, Fu Z, Green JK, Jessica S, Hanan NP, Kokaly RF, Litvak M, Macbean N, Ojima D, Poulter B, Scott RL, Smith WK, Robert J, Tucker CJ, Wang L, Watts J, Wessels K, Zhang F and Zhang W (2024) Adaptation and Response in Drylands (ARID): Community Insights for Scoping a NASA Terrestrial Ecology Field Campaign in Drylands. Earth’s Future 12(9), e2024EF004811. 10.1029/2024EF004811

[r10] Godde CM, Mason-D’ Croz D , Mayberry DE, Thornton PK and Herrero M (2021) Impacts of climate change on the livestock food supply chain; a review of the evidence. Global Food Security 28, 100488. 10.1016/j.gfs.2020.100488 33738188 PMC7938222

[r11] Greene T and Fox K (2021) New NASA Earth System Observatory to Help Address, Mitigate Climate Change. *NASA.* Available at https://www.nasa.gov/news-release/new-nasa-earth-system-observatory-to-help-address-mitigate-climate-change/. Date Accessed: June 1st, 2024.

[r12] Gross N, Maestre FT, Liancourt P, Berdugo M, Martin R, Gozalo B, Ochoa V, Delgado-Baquerizo M, Maire V, Saiz H, Soliveres S, Valencia E, Eldridge DJ, Guirado E, Jabot F, Asensio S, Gaitán JJ, García-Gómez M, Martínez P, Martínez-Valderrama J, Mendoza BJ, Moreno-Jiménez E, Pescador DS, Plaza C, Pijuan IS, Abedi M, Ahumada RJ, Amghar F, Arroyo AI, Bahalkeh K, Bailey L, Ben Salem F, Blaum N, Boldgiv B, Bowker MA, Branquinho C, van den Brink L, Bu C, Canessa R, Castillo-Monroy A d P, Castro H, Castro P, Chibani R, Conceição AA, Darrouzet-Nardi A, Davila YC, Deák B, Donoso DA, Durán J, Espinosa C, Fajardo A, Farzam M, Ferrante D, Franzese J, Fraser L, Gonzalez S, Gusman-Montalvan E, Hernández-Hernández RM, Hölzel N, Huber-Sannwald E, Jadan O, Jeltsch F, Jentsch A, Ju M, Kaseke KF, Kindermann L, le Roux P, Linstädter A, Louw MA, Mabaso M, Maggs-Kölling G, Makhalanyane TP, Issa OM, Manzaneda AJ, Marais E, Margerie P, Hughes FM, Messeder JVS, Mora JP, Moreno G, Munson SM, Nunes A, Oliva G, Oñatibia GR, Peter G, Pueyo Y, Quiroga RE, Ramírez-Iglesias E, Reed SC, Rey PJ, Reyes Gómez VM, Rodríguez A, Rolo V, Rubalcaba JG, Ruppert JC, Sala O, Salah A, Sebei PJ, Stavi I, Stephens C, Teixido AL, Thomas AD, Throop HL, Tielbörger K, Travers S, Undrakhbold S, Val J, Valkó O, Velbert F, Wamiti W, Wang L, Wang D, Wardle GM, Wolff P, Yahdjian L, Yari R, Zaady E, Zeberio JM, Zhang Y, Zhou X, le Bagousse-Pinguet Y, Tielbörger K, Travers S, Undrakhbold S, Val J, Valkó O, Velbert F, Wamiti W, Wang L, Wang D, Wardle GM, Wolff P, Yahdjian L, Yari R, Zaady E, Zeberio JM, Zhang Y, Zhou X and Le Bagousse-Pinguet Y (2024) Unforeseen plant phenotypic diversity in a dry and grazed world. Nature 632(8026), 808–814. 10.1038/s41586-024-07731-3 39112697

[r13] Holmgren M, Stapp P, Dickman CR, Gracia C, Graham S, Gutiérrez JR, Hice C, Jaksic F, Kelt DA, Letnic M, Lima M, López BC, Meserve PL, Milstead WB, Polis GA, Previtali MA, Richter M, Sabaté S and Squeo FA (2006) Extreme climatic events shape arid and semiarid ecosystems. Frontiers in Ecology and the Environment 4(2), 87–95. 10.1890/1540-9295(2006)004[0087:ECESAA]2.0.CO;2

[r31] Huang J, Ji M, Xie Y, Wang S, He Y and Ran J (2016) Global semi-arid climate change over last 60 years. Climate Dynamics 46(3–4), 1131–1150. 10.1007/s00382-015-2636-8

[r14] Jones MO, Naugle DE, Twidwell D, Uden DR, Maestas JD and Allred BW (2020) Beyond Inventories: Emergence of a New Era in Rangeland Monitoring. Rangeland Ecology and Management 73(5), 577–583. 10.1016/j.rama.2020.06.009

[r15] Li F, Kustas WP, Anderson MC, Prueger JH and Scott RL (2008) Effect of remote sensing spatial resolution on interpreting tower-based flux observations. Remote Sensing of Environment 112(2), 337–349. 10.1016/j.rse.2006.11.032

[r16] Loescher HW, Vargas R, Mirtl M, Morris B, Pauw J, Yu X, Kutsch W, Mabee P, Tang J, Ruddell BL, Pulsifer P, Bäck J, Zacharias S, Grant M, Feig G, Zhang L, Waldmann C and Genazzio MA (2022) Building a Global Ecosystem Research Infrastructure to Address Global Grand Challenges for Macrosystem Ecology. Earth’s Future 10(5), 1–11. 10.1029/2020EF001696

[r17] MacBean N, Scott RL, Biederman JA, Peylin P, Kolb T, Litvak ME, Krishnan P, Meyers TP, Arora VK, Bastrikov V, Goll D, Lombardozzi DL, Nabel JEMS, Pongratz J, Sitch S, Walker AP, Zaehle S and Moore DJP (2021) Dynamic global vegetation models underestimate net CO2flux mean and inter-annual variability in dryland ecosystems. Environmental Research Letters 16(9). 10.1088/1748-9326/ac1a38

[r18] Maestre FT, Eldridge DJ, Soliveres S, Kéfi S, Delgado-Baquerizo M, Bowker MA, García-Palacios P, Gaitán J, Gallardo A, Lázaro R and Berdugo M (2016) Structure and Functioning of Dryland Ecosystems in a Changing World. Annual Review of Ecology, Evolution, and Systematics 47(1), 215–237. 10.1146/annurev-ecolsys-121415-032311 PMC5321561 28239303

[r19] Maestre FT, Bagousse-pinguet YL, Delgado-baquerizo M, Eldridge DJ, Saiz H, Berdugo M, Gozalo B, Ochoa V and Guirado E (2022) Grazing and ecosystem service delivery in global drylands. Science 378(6622), 1–7. 10.1126/science.abq4062 36423285

[r20] Miller CE, Griffith PC, Goetz SJ, Hoy EE, Pinto N, McCubbin IB, Thorpe AK, Hofton M, Hodkinson D, Hansen C, Woods J, Larson E, Kasischke ES and Margolis HA (2019) An overview of above airborne campaign data acquisitions and science opportunities. Environmental Research Letters 14(8). 10.1088/1748-9326/ab0d44

[r21] Moore GW, Edgar CB, Vogel JG, Washington-Allen RA, March RG and Zehnder R (2016) Tree mortality from an exceptional drought spanning mesic to semiarid ecoregions. Ecological Applications 26(2), 602–611. 10.1890/15-0330 27209798

[r22] Noy-Meir I (1973) Desert Ecosystems: Environment and Producers. Annual Review of Ecology and Systematics 4(1), 25–51. 10.1146/annurev.es.04.110173.000325

[r23] Poulter B, Frank D, Ciais P, Myneni RB, Andela N, Bi J, Broquet G, Canadell JG, Chevallier F, Liu YY, Running SW, Sitch S and Van Der. Werf GR (2014) Contribution of semi-arid ecosystems to interannual variability of the global carbon cycle. Nature 509(7502), 600–603. 10.1038/nature13376 24847888

[r24] Reed SC, Feldman AF, Hanan NP, Moore DJP, Ojima DS, Smith WK, Wessels K, Amaral C, Babst F, Biederman J, Litvak ME, MacBean N, Poulter B, Scott RL, Babst-Kostecka A, Fu Z, Green JK, Kokaly RF, Swap RJ, Serbin SP, Tucker CJ, Wang L, Watts JD, Wolfe GM, Flores A, Leaf JR, Washington-Allen R, Prentice K, Kachergis E, Reyes J, Ryan J, SanClements M, Loescher H, Leidner A, Swetnam T and Cook B (2025) The ARID Scoping Study Final Report. Oak Ridge, Tennessee: ORNL DAAC.

[r25] Reynolds JF, Stafford Smith DM, Lambin EF, Turner BL, Mortimore M, Batterbury SPJ, Downing TE, Dowlatabadi H, Fernández RJ, Herrick JE, Huber-Sannwald E, Jiang H, Leemans R, Lynam T, Maestre FT, Ayarza M and Walker B (2007) Global desertification: Building a science for dryland development. Science 316(5826), 847–851. 10.1126/science.1131634 17495163

[r26] Safriel U, Adeel Z, Niemeijer D, Puigdefabregas J, White R, Lal R, Winslow M, Prince S, Archer E, King C, Shapiro B, Wessels K, Nielsen T, Portnov B, Reshef I, Lachman E and Mcnab D (2005) Dryland systems. In Ecosystems and Human Well-being: Current State and Trends. (Vol. 1, pp. 623–662). Island Press.

[r27] Schimel DS (2010) Drylands in the earth system. Science 327(5964), 418–419. 10.1126/science.1184946 20093461

[r28] Sitch S, O’Sullivan M, Robertson E, Friedlingstein P, Albergel C, Anthoni P, Arneth A, Arora VK, Bastos A, Bastrikov V, Bellouin N, Canadell JG, Chini L, Ciais P, Falk S, Harris I, Hurtt G, Ito A, Jain AK, Jones MW, Joos F, Kato E, Kennedy D, Klein Goldewijk K, Kluzek E, Knauer J, Lawrence PJ, Lombardozzi D, Melton JR, Nabel JEMS, Pan N, Peylin P, Pongratz J, Poulter B, Rosan TM, Sun Q, Tian H, Walker AP, Weber U, Yuan W, Yue X and Zaehle S (2024) Trends and drivers of terrestrial sources and sinks of carbon dioxide: An overview of the trendy project. Global Biogeochemical Cycles 38(7), 1–25. 10.1029/2024GB008102

[r29] Smith WK, Dannenberg MP, Yan D, Herrmann S, Barnes ML, Barron-Gafford GA, Biederman JA, Ferrenberg S, Fox AM, Hudson A, Knowles JF, MacBean N, Moore DJP, Nagler PL, Reed SC, Rutherford WA, Scott RL, Wang X and Yang J (2019) Remote sensing of dryland ecosystem structure and function: Progress, challenges, and opportunities. Remote Sensing of Environment 233, 111401. 10.1016/j.rse.2019.111401

[r30] St. Germain K, Robinson J, Boukabara S, Wagner T, Seablom M, Sylak-Glassman E, Kaye J, Albers C, Tsaoussi L, Scott J, Becker K, Baynes K, Considine D, Schwinger S, Friedl L, Mihm W, Rogers L, Friedl R, Schwandner F, Dyal T, Ly D, Gasbarre J, Jedlovec G, Kirschbaum D, Neumann T, Walise D, Hain C, Molthan A and Graf J (2024) Earth Science to Action Strategy 2024–2034. Available at https://science.nasa.gov/earth-science/earth-science-to-action/. Date Accessed: February, 1st., 2024

[r32] Tucker C, Brandt M, Hiernaux P, Kariryaa A, Rasmussen K, Small J, Igel C, Reiner F, Melocik K, Meyer J, Sinno S, Romero E, Glennie E, Fitts Y, Morin A, Pinzon J, McClain D, Morin P, Porter C, Loeffler S, Kergoat L, Issoufou BA, Savadogo P, Wigneron JP, Poulter B, Ciais P, Kaufmann R, Myneni R, Saatchi S and Fensholt R (2023) Sub-continental-scale carbon stocks of individual trees in African drylands. Nature 615(7950), 80–86. 10.1038/s41586-022-05653-6 36859581 PMC9977681

[r33] Wang L, Jiao W, MacBean N, Rulli MC, Manzoni S, Vico G and D’Odorico P (2022) Dryland productivity under a changing climate. Nature Climate Change 12(11), 981–994. 10.1038/s41558-022-01499-y

[r34] Watts J, Gennet S, Knapp C, Lavallee J, Ritten J, Hulvey K, Nasto M, Feldman E, Willard S and Toureene C (2025) Grazing Lands Management Practices: An Assessment of Climate Outcomes. *Technical Advisory Network for Climate Smart Agricultural Practices*. https://explore.openaire.eu/search/result?pid=10.5281/zenodo.15750303. Date Accessed: August 1st, 2025.

[r35] Williams AP, Cook BI and Smerdon JE (2022) Rapid intensification of the emerging southwestern North American megadrought in 2020–2021. Nature Climate Change 12(3), 232–234. 10.1038/s41558-022-01290-z

