# Supplementary material for: An overview of the NASA Adaptation and Response in Drylands field experiment scoping study
Source: Camb Prism Drylands. 2026 May 26;3:e22. doi: 10.1017/dry.2025.10006 (PMC13347782; doi:10.1017/dry.2025.10006)
Supplement: Peer review history

# Reviewer report: Review: An overview of the NASA Adaptation and Response in Drylands field experiment scoping study — R0/PR2

**DOI:** 10.1017/dry.2025.10006.pr2

The purpose of this manuscript is to provide a brief overview of the potential NASA Program called Adaptation and Response in Drylands (ARID) based on a 2 year(ish) scoping study. NASA has historically funded intensive and extensive research campaigns in other ecosystems (Boreal, Tropical forest) and the organizers of this scoping study have proposed a similar type of program for drylands. The program would focus intensively on the western US, linking together Ameriflux, LTER, NEON, USDA, BLM, and other acronyms for comprehensive understanding of dryland dynamics. In addition, international sites would be established with partners in South America, Southern Africa and Australia. The goal is to address some big overarching questions centered around climate dynamics, ecosystem structure and biodiversity, carbon cycle, and social-ecological systems.

In truth, there really isn’t much to review here. This is a very high level, 10000 meter summary of goals, locations, and assets to be incorporated in the overall program. Given the impending destruction of the federal research enterprise by the Trump administration, I do not think ARID is going to happen any time soon. But I see two reasons to get this published. First, the information needs to be stored somewhere before the feds hide it. Second, if it is possible to repair the federal research enterprise in the near future, then we need these documents in the public record to restart the initiative. Drylands are vastly understudied and underappreciated, and the overall ARID scoping study does a solid job of highlighting the issues and challenges faced by these globally extensive ecosystems.

I do think the authors need to perhaps more clearly distinguish this manuscript from the paper in Earth’s Future. Secondly, the document is rather dry. Liven it up with some figures, diagrams, photos, or something. A little more effort will go a long way to making this a bit more palatable. Finally, this is pie in the sky writing. ARID will conquer all. It reminds me of documents generated by NSF workshops promoting NEON. Maybe a bit of a nod to limitations so this doesn’t read like an uncritical article in a travel magazine.

I attended several of the outreach events (ESA and AGU) and participated in one of the multiday workshops. I also have a substantial history of research in drylands. So, I am not unbiased in my evaluation. I am a strong proponent of ARID and would like very much to see it happen someday, somehow.

# Editor report: Recommendation: An overview of the NASA Adaptation and Response in Drylands field experiment scoping study — R0/PR3

**DOI:** 10.1017/dry.2025.10006.pr3

**Affiliations:** Arizona State University, United States

Thank you for this submission, please incorporate reviewer’s comments that will imporve the impact of the manuscript.

# Editor report: Decision: An overview of the NASA Adaptation and Response in Drylands field experiment scoping study — R0/PR4

**DOI:** 10.1017/dry.2025.10006.pr4

**Affiliations:** Arizona State University, United States

No accompanying comment.

# Reviewer report: Review: An overview of the NASA Adaptation and Response in Drylands field experiment scoping study — R1/PR6

**DOI:** 10.1017/dry.2025.10006.pr6

The authors have done a nice job of addressing my comments on the original submission. The two new figures help to enliven the document. There are some typos in the new text that should be fixed:

Line 125-126: bedrock is listed twice and defined as, “a photosynthstic (sic) soil community made up of mosses….” I don’t think that is the correct definition of bedrock, but I didn’t bother to google it. Also, please correct the spelling of “photosynthetic.”

Line 144: “…ground-based offer…” A word is missing.

Line 157: “engagement” is misspelled.

Line 260: “scientists” is misspelled!

Line 438: should it be “lays”?

# Editor report: Recommendation: An overview of the NASA Adaptation and Response in Drylands field experiment scoping study — R1/PR7

**DOI:** 10.1017/dry.2025.10006.pr7

**Affiliations:** Arizona State University, United States

Congratulations, your manuscript is accepted. Reviewer found a few typos. Please fix them.

Best

# Editor report: Decision: An overview of the NASA Adaptation and Response in Drylands field experiment scoping study — R1/PR8

**DOI:** 10.1017/dry.2025.10006.pr8

**Affiliations:** Arizona State University, United States

No accompanying comment.
